# The Roles of Small GTPases in Osteoclast Biology

**DOI:** 10.4172/2161-0533.1000161

**Published:** 2014-07-20

**Authors:** Megan M. Weivoda, Merry Jo Oursler

**Affiliations:** Endocrine Research Unit, Mayo Clinic, USA

**Keywords:** Osteoclast, Small GTPase, Rho, Rac, Cdc42, Ras, Podosome, Ruffled border

## Abstract

The adult skeleton undergoes bone remodeling that consists of bone formation by osteoblasts and bone resorption by osteoclasts. When the amount of bone resorbed is greater than the amount of new bone formed, low bone mass results, putting individuals at increased risk for osteoporosis and osteoporotic bone fracture. Nitrogenous bisphosphonates (NBPs) are the most common first line treatment for conditions of low bone mass. NBPs reduce osteoclast bone resorption by impairing the post-translational modification of small GTPases. Small GTPases play crucial roles in the differentiation, function, and survival of osteoclasts. Understanding the roles of individual small GTPases in osteoclast biology may lead to more targeted therapies for the treatment of low bone mass. In this review, we discuss recent investigations into the *in vivo* effects of individual GTPase deletion in osteoclasts and the molecular roles for small GTPases in osteoclast biology.

## Introduction

The adult skeleton undergoes bone remodeling that consists of bone resorption by osteoclasts and subsequent bone formation by osteoblasts. Bone resorption and formation are coupled in order to maintain skeletal mass [1]. Uncoupling of these processes with aging and in certain genetic and environmental conditions causes the amount of bone resorption to exceed bone formation, leading to low bone mass and increased risk for osteoporosis and osteoporotic fractures [2]. Currently, nitrogenous bisphosphonates (NBPs) are the first line treatment for osteoporosis. NBPs reduce bone resorption by inhibiting an enzyme required for the post-translational modification of small GTPases in osteoclasts [3]. NBP-treated patient osteoclasts show features of prolonged apoptosis and resorption markers are significantly decreased [4, 5]. NBPs also reduce the rate of bone formation [6, 7] and there is evidence that these drugs blunt the anabolic effects of parathyroid hormone on the skeleton [8, 9]. The presence of osteoclasts has been shown to be critical to normal bone formation, and it is thought that impaired osteoclast viability is responsible for reduced bone formation with NBP therapy.

In this review we highlight recent investigations into the in vivo effects of individual GTPase deletion in osteoclasts. Additionally, we discuss the molecular roles of small GTPases in regulating osteoclast differentiation, cytoskeletal organization, and vesicular transport. The effectiveness of NBP therapy supports that understanding how small GTPases are modified and their mechanisms of action in regulation of osteoclast function may promote the development of more targeted therapies to suppress bone resorption while preserving the anabolic effects of osteoclasts on osteoblasts.

## Small GTPases

Small GTPases are molecular switches controlling signal transduction [10] and are therefore key regulators of cellular events, including cell division, cytoskeletal organization, and vesicular transport [11]. In basal conditions, GTPases exist in the GDP-bound, inactive state. Guanine-nucleotide Exchange Factors (GEFs) facilitate the release of GDP, allowing the GTPase to bind GTP. GTP-binding causes a conformational change in the GTPase, resulting in activation and interaction with downstream effector proteins. Inactivation of GTPase signaling is dependent on the hydrolysis of the bound GTP to GDP. Small GTPases have low intrinsic ability to hydrolyze GTP. The hydrolysis of GTP is therefore catalyzed by GTPase-Activating Proteins (GAPs) that bind to the active GTPase [12]. The Ras Superfamily of small GTPases can be divided into five subfamilies on the basis of sequence homology: Ras, Rho, Rab, Arf, and Ran. Each subfamily has associated GEFs and GAPs. Additionally, some small GTPase families have Guanine Dissociation Inhibitors (GDIs) that regulate the cytosol versus membrane localization of the GTPases through interactions with prenyl moieties on the small GTPases [13].

Membrane targeting of Ras, Rho, and Rab GTPases is dependent on C-terminal protein prenylation [11]. Prenylation is the post-translational covalent addition of farnesyl or geranylgeranyl lipid moieties to CaaX or CxC motifs on proteins [14], and is catalyzed by the prenyl transferases farnesyl transferase, geranylgeranyl transferase (GGTase)-1 or GGTase-2, otherwise known as Rab GGTase (RabGGTase) [11, 14]. Farnesyl pyrophosphate (FPP) and geranylgeranyl pyrophospate (GGPP), the substrates for farnesylation and geranylgeranylation, respectively, are products of the isoprenoid biosynthesis pathway. By inhibiting enzymes within this pathway, cells can be depleted of these and other isoprenoid metabolites, leading to impaired protein prenylation.

The significance of small GTPases in osteoclast biology came to light when it was discovered that the effect of NBPs to inhibit bone resorption occurred through impaired protein prenylation [15, 16]. NBPs inhibit FPP synthase (FPPS) leading to the depletion of FPP and GGPP, as well as other downstream isoprenoid metabolites. The depletion of these groups impairs the prenylation of GTPases, preventing their membrane localization. Disrupted prenylation of GTPases results in inappropriate signaling, suppressing osteoclast differentiation and function and stimulating apoptosis [17].

## *In vivo* effects of osteoclast small GTPase deletion

Several total and osteoclast-specific small GTPase knockout (KO) mouse models have been analyzed for bone phenotypes. The results from these studies have validated the notion that small GTPases, as well as their GEFs and GAPs, are crucial to proper osteoclast differentiation and function.

Cdc42, a member of the Rho family of small GTPases, was deleted from the osteoclast lineage using the Cathepsin K (Ctsk) promoter to drive Cre recombinase (Cre) expression. The mice exhibited an osteopetrotic phenotype, with an increased ratio of bone volume to total volume (BV/TV), trabecular number (TbN) and trabecular thickness (TbTh). Trabecular spacing (TbSp), osteoclast perimeter, and serum markers of resorption were reduced. The authors showed that the mice exhibited an increase in apoptotic osteoclasts, suggesting that Cdc42 plays a role in osteoclast survival. Consistent with this phenotype, mice with deletion of Cdc42GAP, a Cdc42 regulatory protein, exhibited a decreased bone phenotype, with increased serum markers of resorption, and increased osteoclast area [18].

Rac1 and Rac2 are members of the Rho family of small GTPases. Rac1 is widely expressed, while Rac2 is expressed primarily by the hematopoietic lineage [19]. The effect of Rac2 deletion on the skeleton was first described by Itokowa et al. [20]. Total Rac2 KO mice were of normal size with normal tooth eruption. Similar to the osteoclast-specific Cdc42 KO mice, total Rac2 KO mice exhibited an osteopetrotic bone phenotype, with increased trabecular bone mass and reduced bone resorption. These mice also had increased femoral cortical thickness and lower cortical porosity. In contrast to the osteoclast-specific Cdc42 KO, total Rac2 KO mice exhibited a trend toward increased osteoclast number, which reached significance in male, but not female, mice [20]. A second group validated the *in vivo* role for the Rac GTPases in osteoclast biology; Magalhaes et al., assessed the effect of conditional deletion of Rac1 in osteoclast precursors using the Lysozyme M (LysM) promoter to drive Cre. They also assessed the effect of total Rac2 KO on bone. Osteoclast precursor Rac1 deletion resulted in significant increases in whole body, femoral, and vertebral BMD. These mice also exhibited increased BV/TV and TbN, with decreased TbSp. While intact LysM-Rac1 KO mice did not have altered osteoclast numbers, OVX LysMRac1 KO mice had increased osteoclast numbers compared to OVX controls. Rac2 KO mice showed increased vertebral bone mineral density (BMD), BV/TV, TbN, and decreased TbSp. Osteoclast numbers were unaltered in these mice [21].

In contrast to these Rac KO studies, a third in vivo study found that individual deletion of Rac1 or Rac2 did not yield bone phenotypes. Croke et al., assessed osteoclast-specific Rac1 KO (generated with LysM-Cre), Rac2 total KO, as well as Rac1 and Rac2 double KO (LysM-RacDKO) mice [22]. While deletion of Rac1 or Rac2 alone did not alter the skeletal phenotype, double KO of Rac1 and Rac2 led to an osteopetrotic, high bone mass phenotype. The authors also analyzed the bone phenotype of Ctsk-RacDKO mice, using Ctsk-Cre to delete Rac1 in mature osteoclasts; these mice exhibited an osteopetrotic phenotype. Both the LysM-RacDKO and Ctsk-RacDKO showed increased osteoclast numbers. The authors noted that these osteoclasts were large and irregular, as well as abnormally juxtaposed to the bone [22]. It is not entirely clear what led to the discrepancy in results between the study by Croke et al., and those by Itokowa et al., and Magalhaes et al. One possibility is the age of the animals analyzed. The animals were 8–9 weeks, 12 months, and 7 weeks of age in the studies by Itokowaet al. [20], Magalhaes et al. [21], and Croke et al. [22], respectively. Importantly, all three studies found that deletion of Rac1 and/or Rac2 led to an increased bone phenotype despite normal or increased osteoclast numbers, suggesting that Rac signaling is crucial to the osteoclast resorptive function. Also, in contrast to the blunted anabolic effect of PTH with NBP-mediated global disruption of GTPase signaling [9], deletion of Rac2 augments PTH-induced bone formation [23], suggesting that specific targeting of Rac does not prevent the positive effects of osteoclasts to promote bone formation.

Rap1 is a member of the Ras family of small GTPases. Recently, Zou et al., generated osteoclast specific Rap1 deletion mice, using the Ctsk promoter Cre. Similar to KO of Rac1 and Rac2, Rap1 osteoclast KO mice are osteopetrotic, with high osteoclast numbers, suggesting impaired osteoclast function [24].

The only published Rab family KO model investigated for a bone phenotype is the Rab3D KO model. Rab3D KO mice are osteopetrotic, with increased BV/TV, TbN, and TbTh, and decreased TbSp. Although the number of osteoclasts was unchanged, the percent eroded surface was significantly decreased, suggesting that the high bone mass phenotype was due to impaired bone resorption [25].

The results of these *in vivo* studies demonstrate that targeting the activities of certain GTPases decreases bone resorption and increases bone mass without decreasing osteoclast number; specific targeting of these GTPases in conditions of low bone mass could lead to improved bone formation compared to NBP treatment.

## Functions of small GTPases in osteoclast biology

### Pre-osteoclast proliferation and differentiation

Osteoclasts are multinucleated cells derived from the hematopoietic, myeloid lineage with the unique ability to resorb bone matrix. Differentiation of precursors into mature osteoclasts requires macrophage colony-stimulating factor (M-CSF) and Receptor Activator of NFκB ligand (RANKL) [26]; deletion of these genes in mice leads to severe osteopetrosis [27, 28]. M-CSF binds to the c-Fms receptor and stimulates the proliferation and maturation of pre-cursor cells. M-CSF also plays a role in mature osteoclast survival [29]. Activation of c-Fms leads to expression of RANK, the receptor for RANKL [26]. Signaling through c-Fms and RANK leads to the upregulation and/or activation of the transcription factors microphthalmia-induced transcription factor (MITF) and Nuclear Factor of Activated T-cells cytoplasmic 1 (NFATc1). These transcription factors drive expression of genes necessary for osteoclast function, including Cathepsin K (Ctsk), integrin β3, and tartrate resistant acid phosphatase (TRAP). Activation of RANK in mature osteoclasts promotes cell survival as well as stimulates osteoclastic bone resorption [30]. Small GTPase signaling modulates osteoclast proliferation, differentiation, and survival downstream of M-CSF and RANKL signaling.

**Figure 1:**
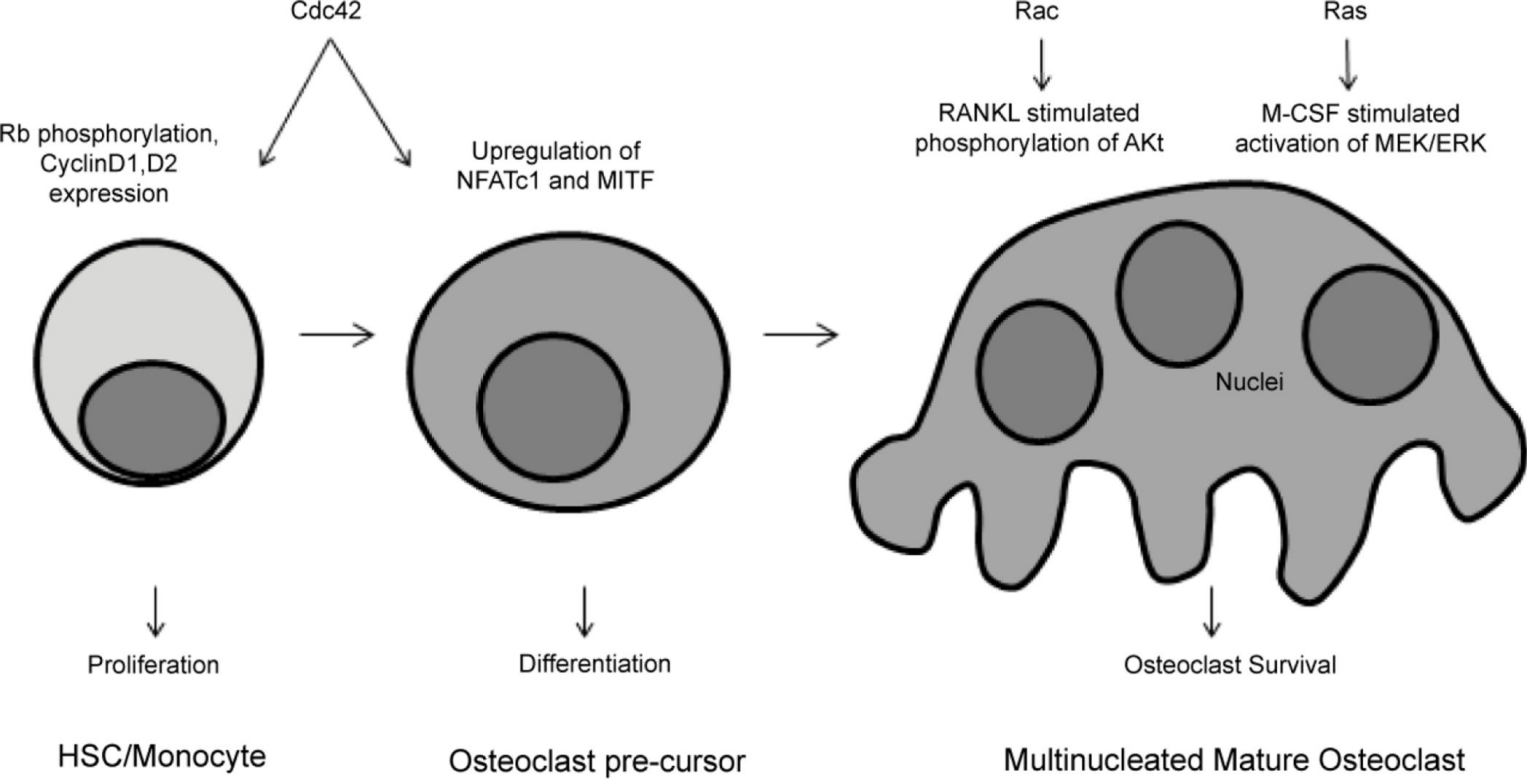
Roles of small GTPases in osteoclast proliferation, differentiation, and survival. Cdc42 promotes the proliferation of osteoclast progenitors as well as expression of MITF and NFATc1 with osteoclast differentiation. Rac and Ras promote osteoclast survival through Akt and MEK/ERK signaling, respectively.

The Rho family small GTPase Cdc42 plays a role in the proliferation, differentiation, and survival of osteoclasts (Figure 1). Cdc42 KO bone marrow osteoclast precursor cultures exhibited impaired M-CSF induced phosphorylation of Rb and expression of Cyclins D1 and D2, leading to reduced proliferation. Knockout of the Cdc42 regulatory protein, Cdc42GAP, resulted in increased proliferation. While Cdc42 KO osteoclast cultures showed reduced differentiation, Cdc42GAP KO cultures showed increased differentiation through the upregulation of NFATc1 and MITF. The Cdc42 KO osteoclasts also exhibited increased apoptosis, consistent with the *in vivo* phenotype [18].

*In vitro* analysis of osteoclasts derived from LysMRacDKO mice, in which both Rac1 and Rac2 are deleted, revealed reduced osteoclast number, in contrast to the *in vivo* phenotype. Osteoclast differentiation was not impaired. Apoptosis was increased in the LysMRacDKO osteoclasts due to impaired pro-survival RANKL-induced Akt phosphorylation at residue Ser 473 (Figure 1) [22].

Ras signaling is important for M-CSF induced osteoclast survival. M-CSF treatment of mature osteoclasts activated Ras and Ras knockdown increased osteoclast apoptosis. Constitutive active Ras promoted osteoclast survival through activation of the MEK/ERK pathway (Figure 1) [29].

### Podosome belt and sealing zone formation

Podosomes are major adhesion structures found in monocyte-derived cells and consist of an F-actin core surrounded by scaffolding proteins [31]. Osteoclasts rely on podosomes to migrate and adhere to bone. Podosomes are found in a clustered pattern in osteoclast precursors. For bone resorption to occur, osteoclasts must reorganize their cytoskeleton [32]. Podosome clusters form rings that expand into a stable podosome belt at the periphery of the mature osteoclast [33]. Attachment to the bone surface causes polarization of osteoclasts, and the podosomes reorganize to form the sealing zone [34]. The sealing zone is a hallmark of resorbing osteoclasts, and separates the resorption lacuna beneath the osteoclast from the bone microenvironment [35].

GTPases are crucial for podosome organization and the assembly of the sealing zone in coordination with integrin signaling [36]. Osteoclasts express the transmembrane integrin-αvβ3 heterodimer. Integrin αvβ3 is activated by “inside-out” signaling, which causes a conformational change in the extracellular region enhancing the affinity for ligand. “Outside-in” integrin signaling is activated by the Arg-Gly-Asp (RGD) amino acid sequence found on bone protein integrin ligands vitronectin, osteopontin, and type I collagen. Ligand bound integrin-αvβ3 stimulates Src, and downstream kinases, modulating the signaling pathways downstream of growth factor and other transmembrane receptors [26]. Knockout of the β3 subunit or Src leads to osteopetrosis in mouse models [37, 38]. Importantly, integrin signaling activates GEFs, such as Vav3, to modulate small GTPase signaling [26].

**Figure 2:**
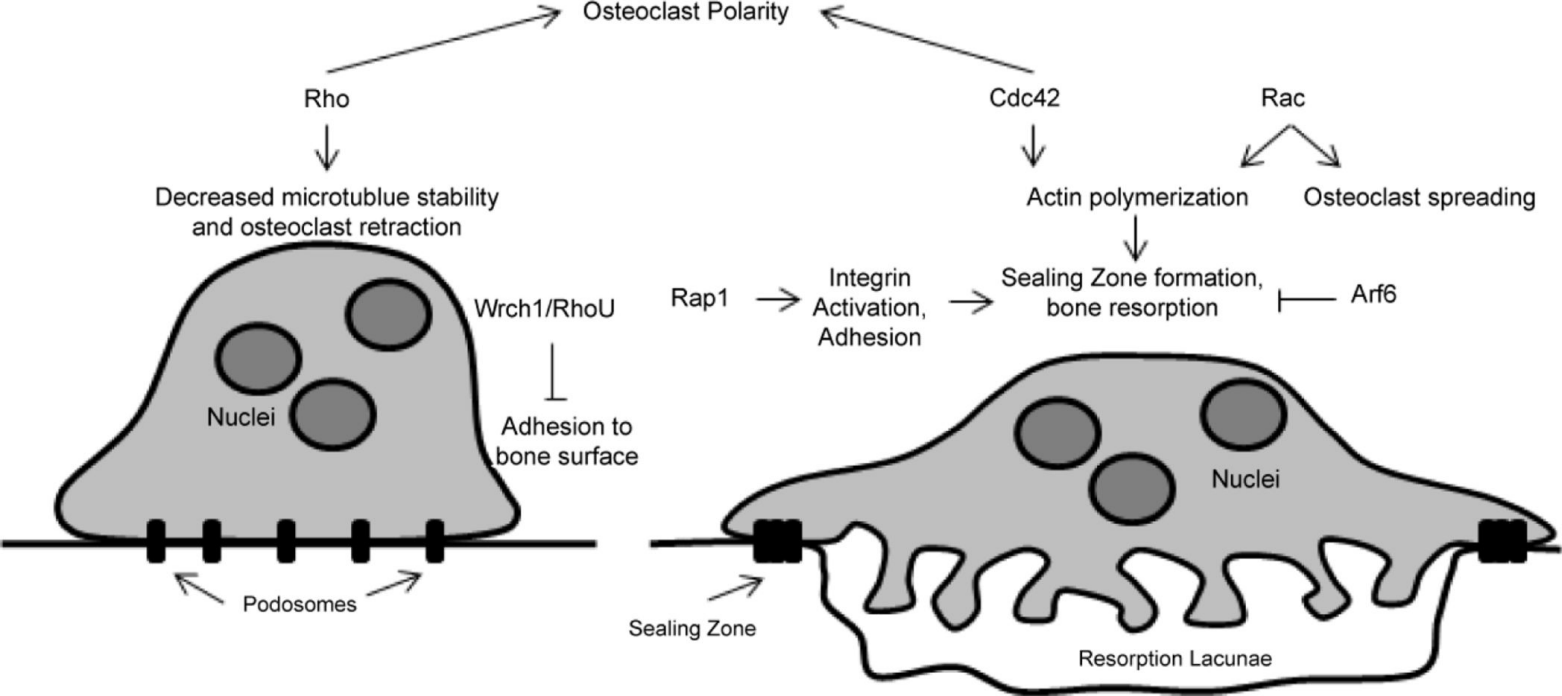
Roles of small GTPases in regulating the cytoskeleton. Rho activity is high in osteoclast precursors and promotes polarization. Rho decreases microtubule stability through activation of mDia/HDAC6 leading to osteoclast retraction. Rap1 promotes the inside-out activation of integrin, causing enhanced affinity for RGD ligand. Affinity for RGD is negatively regulated by Wrch1/RhoU in osteoclast precursors. Rac mediates M-CSF induced osteoclast spreading. Rac and Cdc42 promote actin polymerization and sealing zone formation, which is critical to osteoclast resorption. Arf6 negatively regulates the sealing zone in mature osteoclasts.

The polarized, thicker, and less spread out morphology of osteoclasts on bone correlated with high basal RhoA activity [35]. Inhibition of RhoA, B, and C proteins with Clostridium botulinum C3 exoenzyme disrupted the ringed structure of podosomes [39] and impaired osteoclast polarization on apatite-coated slides (Figure 2) [35]. Conversely, injection of mouse bone marrow-derived osteoclasts with activated RhoA or treatment with osteopontin (OPN) to induce RhoA activation caused podosomes to disappear or be redistributed away from cell periphery [40]. Activation of Rho resulted in osteoclast retraction [41], and correlative results were obtained in multinucleated giant cell (MNGC) avian osteoclast-like cultures, in which Rho inhibition caused cell spreading, and activation of Rho caused cell retraction [42]. Together these data suggest that that the cytoskeletal architecture in osteoclasts is tightly regulated by Rho activity.

Rho activation regulates podosome organization in part through regulation of microtubule acetylation. Podosome belt formation in osteoclasts correlated with increased microtubule acetylation. Destaing et al., found that Rho acts through its effector mDia2 to activate HDAC6-mediated deacetylation of microtubules. Nocodazole, an anti-neoplastic agent that causes microtubule depolymeraization, led to podosome belt disruption. Inhibition of Rho delayed nocodazole-induced podosome belt disruption by decreasing microtubule deacetylation and, therefore, increasing podosome stability [43].

Active Rac1 colocalized with actin ruffles as well as the periphery of the osteoclast. Microinjection of active Rac1 led to flattening and spreading of MNGCs, whereas dominant negative Rac1 caused cell retraction, with vinculin aggregation and F-actin disorganization [42]. In a similar study, microinjection of active Rac, but not active Cdc42 or RhoA, mimicked the effects of M-CSF to induce cell spreading. M-CSF induced spreading was impaired with dominant negative Rac, but not dominant negative Cdc42 or inhibition of Rho [41]. Razzouk et al., found that inhibition of Rac1 or 2 with anti-Rac antibodies disrupted actin ring formation, reduced osteoclast resorption and caused retraction of osteoclasts [44]. DN-Rac1-mediated cell retraction did not occur when Rho was inhibited simultaneously. This data suggest that Rac and Rho have antagonistic functions in the regulation of the osteoclast cytoskeleton (Figure 2) [42]. Osteoclasts generated from Rac2 KO mice exhibited reduced actin ring formation and abnormal actin accumulation *in vitro* [20]. While Croke et al., did not find an effect of deleting either Rac1 or Rac2 alone, osteoclasts generated from Rac1 and Rac2 double KO (LysM-RacDKO) mice failed to spread, similar to cells lacking integrin signaling molecules [22]. In a study by Goldberg et al., while knocking down Rac1 versus Rac2 individually led to an equal reduction in bone resorption *in vitro*, Rac1 knockdown reduced the surface area of osteoclasts, whereas Rac2 knockdown did not, suggesting that Rac1 and Rac2 may have specific activities in regulating the osteoclast cytoskeleton [32].

Osteoclasts generated from Cdc42 KO mice displayed reduced actin ring formation and reduced bone resorption *in vitro* ; the opposite was seen in osteoclasts lacking the Cdc42 regulatory GAP, Cdc42GAP [18]. Chellaiah et al., reported that Cdc42 enhanced Rho-induced actin ring formation by augmenting the interaction of Wiscott-Aldrich syndrome protein (WASP) with Arp2/3, which is important for actin nucleation and polymerization [40]. Ito et al., also provide evidence that Cdc42 may be involved in maintaining osteoclast polarity through interactions with Par-3, Par-6, and atypical protein kinase C (aPKC) [18], similar to a mechanism maintaining cell polarity in epithelial cells [45].

Deletion of Rap1, a member of the Ras subfamily of GTPases, led to a crenated appearance and reduced resorbing capacity of osteoclasts *in vitro*. This phenotype was similar to that of osteoclasts lacking integrin signaling molecules. In contrast to the roles of the Rho subfamily of small GTPases downstream of “outside-in” integrin activation, Rap1 is important for “inside-out” integrin activation. Rap1 and its binding partner, Rap1 Interacting Adaptor Molecule (RIAM), activate talin1 to allow interaction with β integrins, increasing the affinity of β integrins for ligand. Deletion of Rap1, therefore, disrupted the ability of integrins to activate cytoskeletal organization (Figure 2) [24].

Arf6 is a member of the Arf subfamily of small GTPases. Overexpression or constitutive-active expression of Arf6 impaired sealing zone formation in osteoclasts. The Arf6 GAP, GIT2, localized to the sealing zone of mature osteoclasts in a Src-dependent manner, and GIT2 knockdown impaired actin ring formation. These results demonstrated that Src-mediated repression of Arf6 is required to maintain the sealing zone in mature, bone-resorbing osteoclasts (Figure 2) [46].

Wrch/RhoU localized to the adhesion structures in osteoclasts [47]. Expression of wild type and constitutive-active Wrch1/RhoU increased the fraction of osteoclasts exhibiting podosomes in clusters/rings rather than in the podosome belt conformation, suggesting that Wrch1/RhoU may negatively regulate podosome belt formation. Wild type and active Wrch1/RhoU inhibited the adhesion of osteoclast precursors to vitronectin (Figure 2). While Wrch1/RhoU associated with the sealing zone, expression did not affect bone resorption [48].

### Vesicular trafficking and the Ruffled Border

Following cytoskeletal reorganization to form the sealing zone, the bone-apposed membrane within the sealing zone becomes highly convoluted by polarized vesicular transport; this membrane domain is called the ruffled border [49]. The ruffled border is crucial to the ability of osteoclasts to resorb bone, and is referred to as the “resorption organelle.” Unlike conventional plasma membranes, the ruffled border is made up of proteins associated with the endosomal/lysosomal membrane, including LAMP1, LAMP2, lgp110, Rab7 and vacuolar H^+^, ATPase (V-ATPase) [49, 50]. The ruffled border membrane is formed by fusion of lysosomes with the plasma membrane that is juxtaposed to the bone surface. A marker of early endosomes, EEA1, is restricted to the osteoclast cytoplasm, and is not present at the ruffled border [51].

When lysosomes fuse with the nascent ruffled border, they release their acidic contents into the resorption lacuna [50, 52]. This fusion delivers membrane-associated proteins important to the resorption process, The V-ATPase and the ClC7 H+/Cl− antiporter (ClC7). VA-TPase acidifies the resorption lacuna, while ClC7 maintains electroneutrality. CTSK is delivered to the lacunae to mediate degradation of the organic collagen matrix [49].

Rab proteins are distributed to distinct intracellular compartments and regulate transport between organelles [53]. The C-terminus of the Rab proteins possesses a CAAX box which is covalently modified with one or two geranylgeranyl prenylation groups by GGTase2 (RabGGTase). This prenylation modification is important to the membrane localization and, therefore, the function of the Rab family members [11].

The gunmetal KO mouse has a mutation in RGGT that causes a 75% loss of enzyme activity, leading to reduced prenylation of Rab proteins in several cell types, including osteoclasts. Taylor et al., showed reduced prenylation of Rab2B, Rab3D, Rab5, Rab6, Rab7 and Rab14, specifically in the gunmetal KO osteoclasts. The gunmetal KO bone marrow osteoclast cultures exhibited normal differentiation and polarization, but reduced resorptive activity *in vitro*, demonstrating the importance of the Rab proteins to the osteoclast resorptive function [54]. Consistent with this, disruption of Rab geranylgeranylation with a phosphonocarboxylate inhibitor of RabGGTase inhibited osteoclast bone resorption [55]. In contrast to the expected osteopetrotic phenotype due to impaired osteoclast resorption, the gunmetal KO mice have osteopenia, suggesting that defects in other cell types contribute to the bone phenotype [54], or that Rab proteins contribute to an osteoclast secretory function necessary for coupling of bone formation to bone resorption.

**Figure 3:**
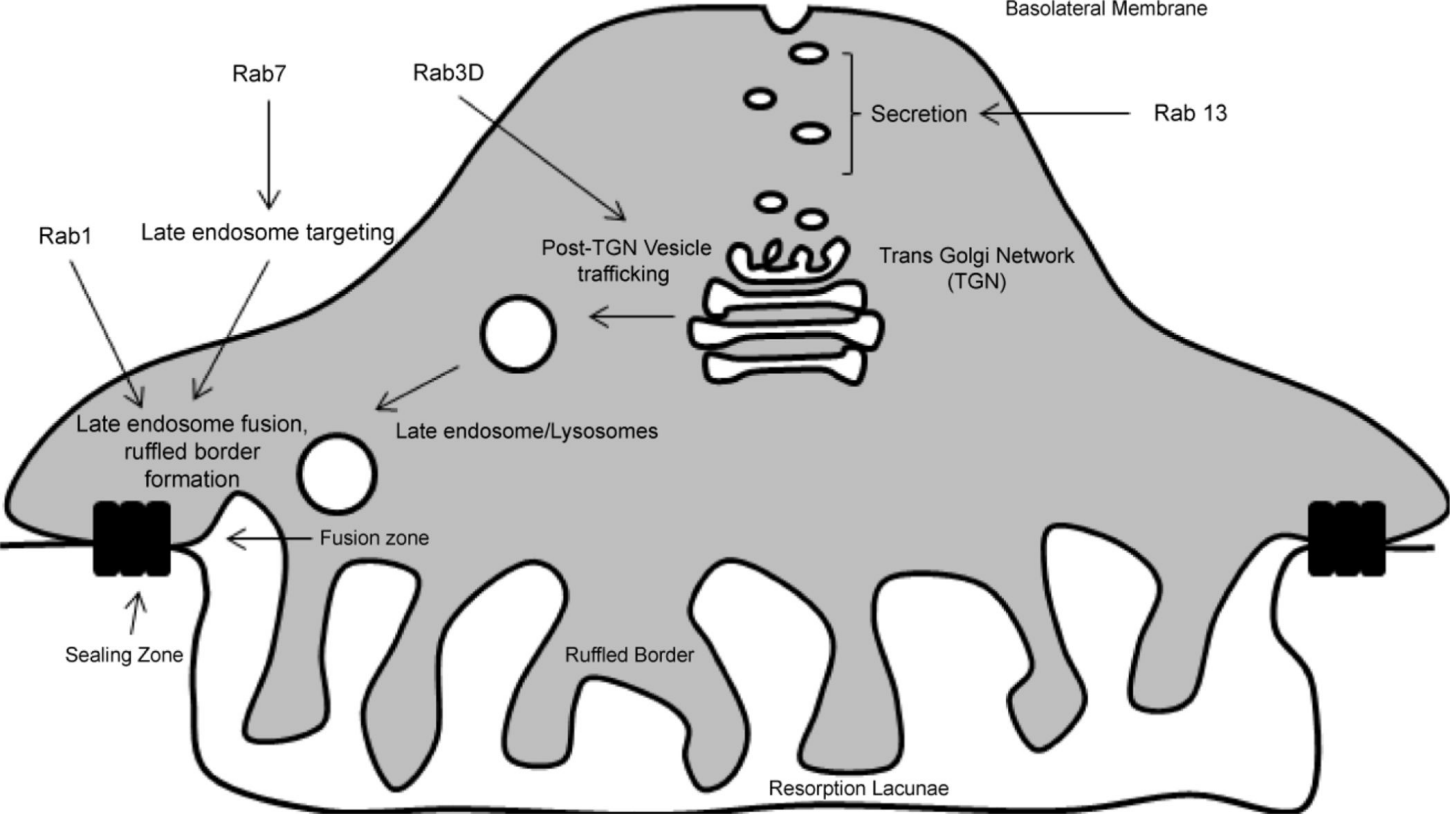
Roles of small GTPases in vesicular trafficking. Rab3D localizes to the lysosomal subset of the Trans Golgi Network (TGN) and is necessary for vesicular trafficking to the ruffled border. Rab7 localizes with late endosomes/lysosomes and the ruffled border. Rab7 and Rac1 are thought to facilitate the fusion of vesicles to the ruffled border. Rab13 localizes to small vesicles between the TGN and basolateral membrane and is thought to be involved in secretion.

There are more than 60 Rab family members in the human genome [53]. Using a variety of methods, it has been shown that the osteoclast lineage express several Rab family members, including Rab1A, Rab1B, Rab2B, Rab3D, Rab4B, Rab5C, Rab6, Rab7, Rab9, Rab11B, Rab14, Rab 18, and Rab35 [51, 54]. Using microscopy, Zhao et al., showed that Rab5C associated with early endosomes, and Rab11B localized to the perinuclear recycling compartments. The late-endosomal Rab7 and Rab9 co-localized around the nuclei. Rab7 was the only Rab family member to localize to the ruffled border. The similar distribution of Rab7 and V-ATPase suggest that Rab7 regulates the targeting and fusion of the late endosomes/lysosomes to the ruffled border [51]. Knockdown of Rab7 disrupted the distribution of V-ATPase to the ruffled border, impaired ruffled border formation, and inhibited bone resorption *in vitro* (Figure 3) [56].

A recent publication demonstrated that autophagy proteins Atg5, Atg7, Atg4b, and LC3 have a role in the polarized transport of lysosomes to the resorption lacunae and are therefore important for the generation of the osteoclast ruffled border. Knockdown of Atg5 reduced the localization of Rab7, CTSK, LAMP1 to the ruffled border and reduced bone resorption *in vitro* and *in vivo* [57].

As discussed in the previous section, Rac1 plays a critical role in the cytoskeletal organization of osteoclasts. Rac1 may also have a function in establishment of the osteoclast ruffled border. Sun et al., demonstrated that Rab7 colocalized with Rac1 at the fusion zone of the ruffled border in osteoclasts cultured on bovine bone. Because of the role of Rac1 in the regulation of the actin cytoskeleton, the authors proposed that a Rab7-Rac1 interaction may mediate the transport of late endosomes between microtubules and microfilaments. This interaction may subsequently regulate the osteoclast ruffled border formation (Figure 3) [58].

Rab3D is the major Rab3 isoform found in precursor and mature osteoclasts. Rab3D KO mouse osteoclasts have impaired bone resorption *in vitro*, consistent with the decreased eroded surface of the bone in vivo. Pavlos et al., demonstrated that Rab3D deficient osteoclasts had irregular ruffled borders. Wild type and constitutive-active Rab3D localized to the nonendosomal/lysosomal subset of post-Trans-Golgi-Network (TGN). Interestingly, a dominant negative Rab3D mutant inhibited the biogenesis of these vesicles. These results suggest that Rab3D is involved in the regulation of a post-TGN vesicle trafficking step that is required for the maintenance of the osteoclast ruffled border and bone resorption (Figure 3) [25].

Recently, Rab13 was shown to be upregulated during the osteoclast differentiation of human peripheral blood mononuclear cells (PBMCs). Rab13 localized to small vesicles between the osteoclast TGN and basolateral membrane, and the authors suggest that Rab13 may play a role with a putative secretory function in osteoclasts (Figure 3). However, siRNA-mediated knockdown of Rab13 in osteoclasts did not lead to significant effects *in vitro* [59]. It would be of interest to determine the effects of Rab13 on osteoclast protein secretion and osteoblast coupling.

## Concluding Remarks

There is substantial evidence that small GTPases are crucial to osteoclast function. NBPs have proven to be an effective therapy to reduce osteoclast bone resorption *in vivo* is limited by a significant reduction in bone formation. Further understanding of the individual roles of GTPases in osteoclast biology may allow for the development of osteoporosis therapies that selectively impair bone resorption without disrupting osteoclast viability and the anabolic effects of osteoclasts on osteoblasts.
